# Ataxia-telangiectasia mutated activation mediates tumor necrosis factor-alpha induced MMP-13 up-regulation and metastasis in lung cancer cells

**DOI:** 10.18632/oncotarget.11386

**Published:** 2016-08-19

**Authors:** Hong Qiong Yan, Di Zhang, Yuan Yuan Shi, Xiang You, Lei Shi, Qing Li, Feng Guang Gao

**Affiliations:** ^1^ Department of Immunology, Basic Medicine Science, Medical College, Xiamen University, Xiamen 361102, People's Republic of China; ^2^ State Key Laboratory of Oncogenes and Related Genes, Shang Hai Jiao Tong University, Shanghai, 200032, People's Republic of China

**Keywords:** tumor necrosis factor-alpha, ataxia-telangiectasia mutated, lung cancer, migration, matrix metalloproteinases

## Abstract

Despite that ataxia-telangiectasia mutated (ATM) is involved in IL-6 promoted lung cancer chemotherapeutic resistance and metastasis, the exact role of ATM in tumor necrosis factor-alpha (TNF-α) increasing tumor migration is still elusive. In the present study, we demonstrated that TNF-α promoted lung cancer cell migration by up-regulation of matrix metalloproteinase-13 (MMP-13). Notably, by gene silencing or kinase inhibition, we proposed for the first time that ATM is a key up-stream regulator of TNF-α activated ERK/p38-NF-κB pathway. The existence of TNF-α secreted in autocrine or paracrine manner by components of tumor microenvironment highlights the significance of TNF-α in inflammation-associated tumor metastasis. Importantly, *in vivo* lung cancer metastasis test showed that ATM depletion actually reduce the number of metastatic nodules and cancer nests in lung tissues, verifying the critical role of ATM in metastasis. In conclusion, our findings demonstrate that ATM, which could be activated by lung cancer-associated TNF-α, up-regulate MMP-13 expression and thereby augment tumor metastasis. Therefore, ATM might be a promising target for prevention of inflammation-associated lung cancer metastasis.

## INTRODUCTION

Tumor necrosis factor-alpha (TNF-α), a vital pro-inflammatory cytokine in tumor microenvironment (TME) [1], acts as an endogenous tumor promoter to facilitate invasion and metastasis [2–3]. When TNF-α binds to its receptors, TNF-α could recruit intracellular adaptor proteins and NF-κB, JNK, p38 MAPK, ERK, AP-1 or PI3K pathway was activated [4]. As a key coordinator of innate immunity [5], NF-κB was involved in TNF-α receptor signaling and up-regulate its downstream effectors [6]. While NF-κB inhibition decreased TNF-α-induced invasion [7–10], ATM was also documented to be a novel upstream regulator of IL-6 induced NF-κB activation [11–12]. But, the exact role of ATM in TNF-α promoted metastasis and the mechanism by which ATM mediating TNF-α induced NF-κB activation is still uncertain.

MMPs, such as MMP-3, MMP-9, MMP-13 were negative prognostic factors for lung cancer survival [13–18]. MMP-13, which could be activated by MMP-2, MMP-3 and MMP-14 [19], functionally degraded type II collagen, resulting in extracellular matrix (ECM) turnover [20–21]. Activated MMP-13 also had positive effect on MMP-2 and MMP-9 activation [21]. Our previous studies showed that MMP-13 mediate IL-6 increasing lung cancer metastasis via ATM activation [11], therefore, we speculate that MMP-13 might be a critical potential target for TNF-α augmenting lung cancer migration.

Here, we found that TNF-α promote lung cancer metastasis by MMP-13 up-regulation and ATM activation. ATM inhibition largely decrease TNF-α augmented lung cancer cell migration. The existence of TNF-α secreted in autocrine or paracrine manner by component of tumor microenvironment highlights the significance of TNF-α in inflammation-associated tumor metastasis. Importantly, *in vivo* lung cancer metastasis test showed that ATM depletion actually reduce the number of metastatic nodules and cancer nests in lung tissues, verifying the critical role of ATM in lung cancer metastasis. Therefore, ATM might be a promising target for prevention of inflammation-associated lung cancer metastasis.

## RESULTS

### TNF-α level has a positive correlation with cell migration in lung cancer cells

**Figure 1:**
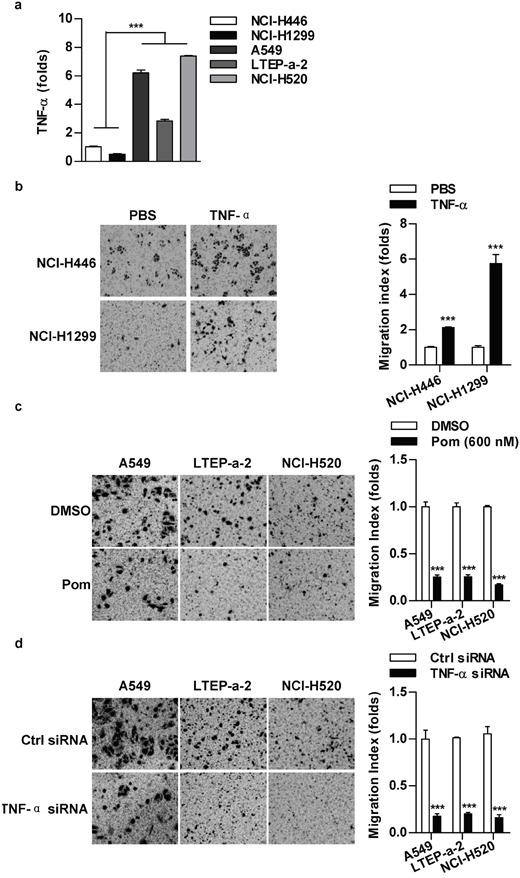
TNF-α level has a positive correlation with cell migration in lung cancer cells **a.** TNF-α level in lung cancer cells was determined by RT-qPCR. **b-d.** TNF-α level in indicated cells was regulated by TNF-α (2 ng/ml) appending (b), TNF-α inhibition (Pom, 600 nM) (c) or siRNA transfection (d) and cell migration was determined via Transwell migration assay. Data are presented as the mean±SEM, n=3. ***p<0.001, Student *t* test or one-way ANOVA with post Newman-Keuls test. One representative from three experiments is shown. Pom: Pomalidomide.

A549, LTEP-a-2 and NCI-H520 cells possess stronger migration abilities than NCI-H446 and NCI-H1299 cells [11]. To explore the effect of TNF-α on cell migration, we firstly determined TNF-α level in a panel of lung cancer cells. As predicted, the higher TNF-α level was revealed in A549, LTEP-a-2 and NCI-H520 cells (Figure 1a). When NCI-H446 or NCI-H1299 cells were replenished with TNF-α, the migration abilities increased accordingly (Figure 1b). A significant repression of cell migration of A549, LTEP-a-2 and NCI-H520 cells was achieved when TNF-α was inhibited by inhibitor (Figure 1c) or gene silencing (Figure 1d). As cell viabilities had not been affected by above treatments (Supplementary Figure S1), our data demonstrate that there is a positive correlation between TNF-α level and cell migration in lung cancer cells.

### MMP-13 is involved in TNF-α promoting lung cancer cell migration

**Figure 2:**
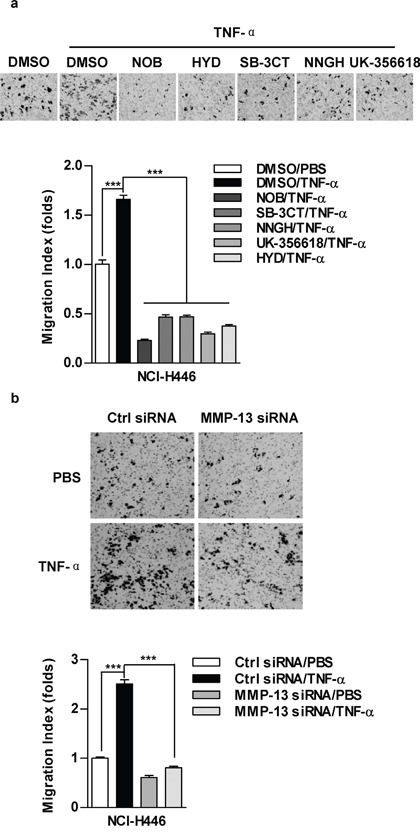
MMP-13 is involved in TNF-α increasing cell migration in lung cancer cells NCI-H446 cells were pretreated with MMPs inhibitors NOB (64 μM), HYD (50 μM), SB-3CT (10 μM), NNGH (10 μM), UK-356618 (73 nM) **a.** or MMP-13 silencing **b.** prior to TNF-α (2 ng/ml) stimulation. Cell migration was determined by Transwell migration assay. Data are presented as the mean±SEM, n=3. ***p<0.001, One-way ANOVA with post Newman-Keuls test. One representative from three experiments is shown. NOB: Nobiletin; HYD: N-CBZ-Pro-Leu-Gly hydroxamate.

Broad-spectrum inhibitor (NOB) and specific inhibitors, including HYD (MMP-1), SB-3CT (MMP-2), NNGH (MMP-3) and UK-356618 (MMP-13), efficiently abolished the effect of TNF-α on cell migration in NCI-H446 cells (Figure 2a). As MMP-13 is closely involved in IL-6 or TNF-α increasing tumor metastasis [11, 22], we therefore focused on MMP-13 in TNF-α increasing lung cancer cell migration. Our results showed that MMP-13 deficiency abrogate TNF-α effect on cell migration (Figure 2b). Additionally, the inhibition of MMP-3 or MMP-9 could achieve similar results in NCI-H446 and A549 cells (Supplementary Figure S2), confirming the crucial role of MMPs in TNF-α promoting lung cancer cell migration.

### TNF-α up-regulate the expression and the activity of MMP-13

**Figure 3:**
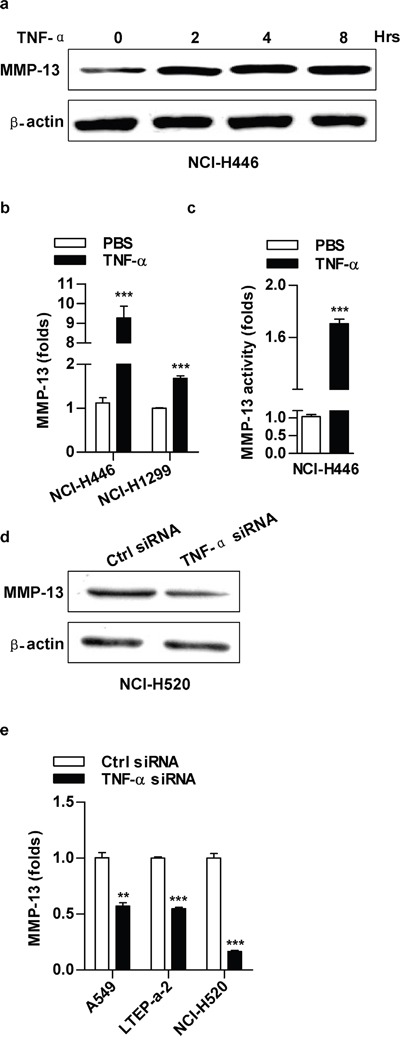
TNF-α increase MMP-13 expression and activity in lung cancer cells Cells were treated with TNF-α (2 ng/ml) **a-c.** or TNF-α silencing **d-e.** and MMP-13 expression (a-b, d-e) or activity (c) was determined by western blot (a, d), RT-qPCR (b, e) or ELISA (c), respectively. Data are presented as the mean±SEM, n=3. **p<0.01, ***p<0.001, Student *t* test. One representative from three experiments is shown. The immunoblots were cropped to improve the clarity and conciseness of the presentation.

Since there is a difference of MMP-13 in expression and activity between NCI-H446 and A549 cells (Supplementary Figure S3a), we therefore explored TNF-α's effect on MMP-13 expression. As shown in Figure 3, TNF-α potently increased MMP-13 expression in both protein (Figure 3a) and mRNA (Figure 3b) level. MMP-13 activity was also increased upon TNF-α's stimulation (Figure 3c). Moreover, TNF-α depletion achieved a significant MMP-13 reduction (Figure 3d-3e). Apart from MMP-13, the expression or activity of MMP-1, MMP-2, MMP-3 and MMP-9 were increased by TNF-α treatment as well (Supplementary Figure S3b-S3d). Collectively, above data support that TNF-α promote cell migration by up-regulation of MMPs expression and activity.

### ATM is involved in TNF-α inducing ERK/p38-NF-κB pathway activation in lung cancer cells

**Figure 4:**
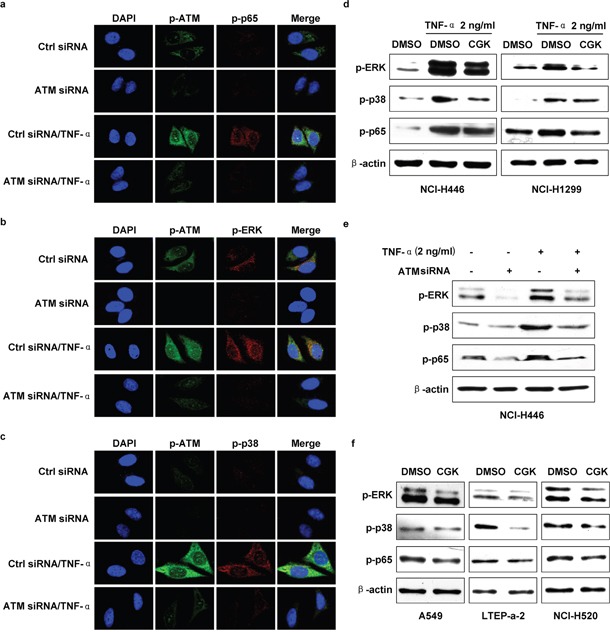
ATM mediate TNF-α inducing ERK/p38-NF-κB activation in lung cancer cells Cells were conferred ATM silencing **a-c, e.** or CGK733 treatment **d, f.** prior to TNF-α (2 ng/ml) stimulation and the phosphorylation of ERK, p38 or p65 was investigated by confocal fluorescence microscope (a-c) or western blot (d-f). One representative from three experiments is shown. CGK: CGK733. The immunoblots were cropped to improve the clarity and conciseness of the presentation.

Owing to TNF-α' effects on ATM, ERK, p38 and p65 activation (Supplementary Figure S4a-S4d), the inhibition of ERK or p38 decreasing TNF-α induced p65 activation (Supplementary Figure S4e) indicate that ATM might play a potential role in TNF-α induced ERK/p38-NF-κB activation. To address this issue, ATM inhibition was performed and TNF-α induced ERK/p38-NF-κB activation was evaluated. As showed in Figure 4a, ATM deficiency abrogated TNF-α's effect on p65 activation. Likewise, TNF-α induced phosphorylation of ERK (Figure 4b, Supplementary Figure S5a) and p38 (Figure 4c, Supplementary Figure S5b) was also abolished by the deficiency of ATM. Western blot assay testified that depletion of ATM by inhibitor (Figure 4d) or gene silencing (Figure 4e) decreased TNF-α induced the activation of ERK, p38 and p65. Furthermore, ATM inhibition directly blocked the phosphorylation of ERK, p38 and p65 (Figure 4f). The above observations indicate that ATM phosphorylation is a key upstream event in regulating TNF-α mediated ERK/p38-NF-κB activation.

### TNF-α up-regulate MMP-13 expression and promote cell migration via ATM-ERK/p38-NF-κB pathway

**Figure 5:**
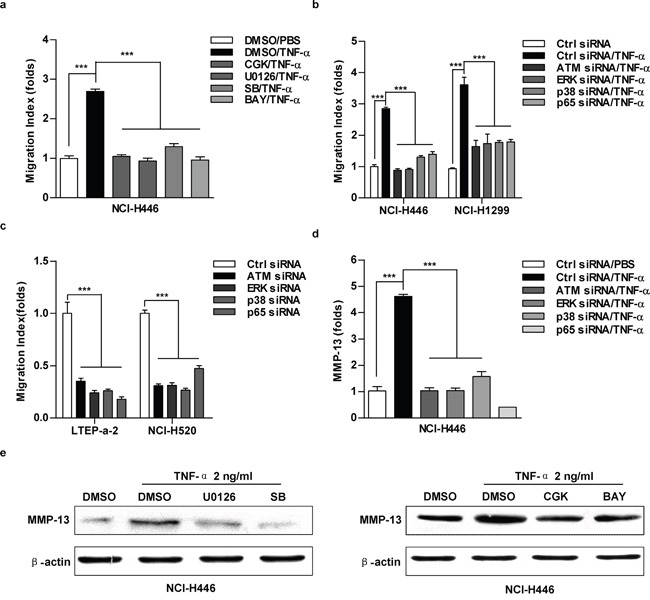
The inhibition of ATM-ERK/p38-NF-κB abrogate TNF-α increasing MMP-13 expression and cell migration in lung cancer cells Cells were pretreated with CGK733 (20 μM), U0126 (20 μM), SB203580 (20 μM), BAY11-7082 (10 μM) **a, e.** or indicated silencing **b-d.** prior to TNF-α (2 ng/ml) stimulation. Cell migration and MMP-13 expression were determined by Transwell migration assay (a-c), RT-qPCR (d) or western blot (e), respectively. Data are presented as the mean±SEM, n=3. ***p<0.001, One-way ANOVA with post Newman-Keuls test. One representative from three experiments is shown. CGK:CGK733; SB:SB203580; BAY: BAY11-7082. The immunoblots were cropped to improve the clarity and conciseness of the presentation.

As ATM-NF-κB pathway is involved in IL-6-increased cell migration [11], we next explored the role of ATM-ERK/p38-NF-κB activation in TNF-α promoting lung cancer cell migration. The repression of ATM-ERK/p38-NF-κB pathway by kinase inhibitors (Figure 5a, Supplementary Figure S6a) or gene silencing (Figure 5b, Supplementary Figure S6b) eliminated the effect of TNF-α on cell migration. ATM-ERK/p38-NF-κB depletion also decreased cell migration in the cells which have higher TNF-α level (Figure 5c, Supplementary Figure S6c). Importantly, TNF-α increasing MMP-13 expression was reversed by the inhibition of ATM-ERK/p38-NF-κB (Figure 5d-5e). A similar reduction of MMP-2/MMP-3 was observed at the comparable condition (Supplementary Figure S6d-S6e). Together, these results demonstrate that ATM-ERK/p38-NF-κB play important roles in TNF-α up-regulating MMP-13 expression and promoting lung cancer cell migration.

### The components of lung cancer microenvironment could secret TNF-α in autocrine or paracrine manner upon LPS or chemotherapeutic drug stimulation

**Figure 6:**
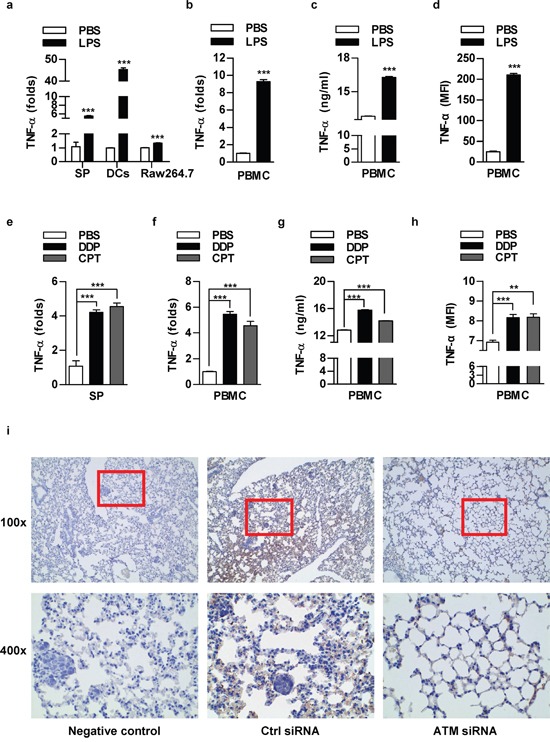
The components of lung cancer microenvironment could secret TNF-α in autocrine or paracrine manner upon LPS or chemotherapeutic drug stimulation Murine splenocytes, dendritic cells, Raw264.7 cells **a, e.** or human peripheral blood mononuclear cells (PBMC) **b-d, f-h.** was stimulated with LPS (a-d) or treated with DDP (1 μg/ml)/CPT (2 μg/ml) (e-h) and TNF-α expression was determined by RT-qPCR (a-b, e-f), ELISA (c, g) and flow cytometric analyse (d, h). The concentration of LPS for splenocytes, PBMC, Raw264.7, DCs was 100, 50, 50, 20 ng/ml respectively. Data are presented as the mean±SEM, n=3. **p<0.01; ***p<0.001, Student *t* test or one-way ANOVA with post Newman-Keuls test. One representative from three experiments is shown. SP: Splenocytes; DCs: dendritic cells. **i.** 8×10^5^ NCI-H520 cells conferred ATM silencing were transferred to BALB/c nude mice (5-6 weeks old) through tail vein (n=3 per group) and the lungs were performed TNF-α IHC staining.

As main components of tumor microenvironment, both mouse and human immune cells efficiently produce TNF-α in low concentration of LPS (Figure 6a-6d) or chemotherapeutic agents (Figure 6e-6h) treated condition. TNF-α IHC determination of lung cancer metastasis test showed that TNF-α expression in cancer nests and surrounding tissues was decreased by the depletion of ATM (Figure 6i). Other groups also found that TNF-α was highly expressed in human non-small cell lung carcinoma [23–24]. These results suggest that TNF-α might be an important component in tumor microenvironment to trigger inflammation associated tumor metastasis.

### ATM inhibition decrease lung cancer metastasis *in vivo*

**Figure 7:**
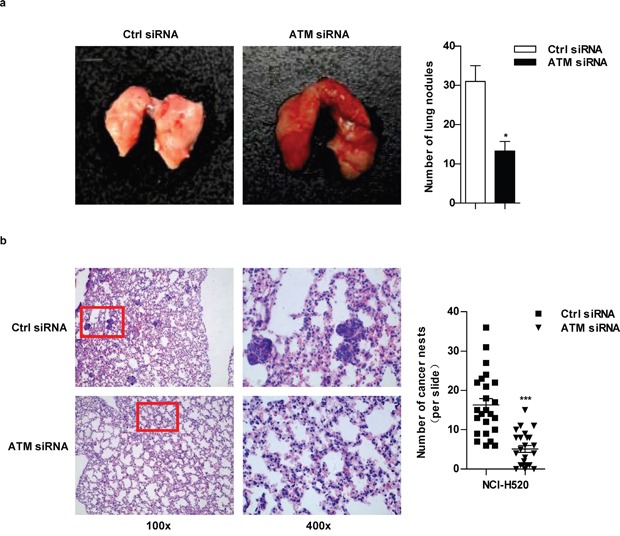
ATM inhibition decrease lung cancer metastasis *In vivo* lung cancer metastasis model was established as described in Figure 6i. After sacrifice, the intact mouse lungs were dissociated to count the white-tan nodules of metastatic tumor by naked eyes **a.** and then embedded to perform H&E staining **b.** For H&E staining, the number of cancer nests in lungs was counted under microscope with 100×magnification; 24 slides per condition (b).

Tail-vein-injected mouse model was used to assess lung cancer metastasis *in vivo* [25]. As shown in Figure 7a, the number of white-tan nodules of metastatic tumor counted by naked eyes dropped when ATM was knocked down. Hematoxylin and eosin staining in Figure 7b showed that: a large number of normal alveolar structures were destroyed and replaced by tumor cells with prominent and irregular nuclei, more cancer nests were observed in control group. Conversely, more normal alveolar structures and a significant drop of the number of cancer nests were found in ATM depletion condition. These observations confirm that high level of ATM promote lung cancer metastasis *in vivo*.

## DISCUSSION

TNF-α and IL-6 are the major pro-inflammatory cytokines to promote metastasis [11, 22, 26–29]. In the present study, we demonstrated that the activation of ATM-ERK/p38-NF-κB, which induced by TNF-α in autocrine or paracrine manner, up-regulate MMP-13 expression and thereby augment lung cancer metastasis. Most importantly, ATM inhibition potently repress the lung cancer metastasis *in vivo*. Therefore, ATM might be a promising target for prevention of inflammation-associated lung cancer metastasis.

TNF-α, which expressed by the components of tumor microenvironment, was functionally controlled by other pro-inflammatory cytokines in paracrine manner [30]. In our observation, TNF-α level has a positive correlation with metastasis of lung cancer, indicating that tumor cells itself might regulate metastasis in autocrine manner. As lung carcinoma epithelium cell with K-ras mutation have higher level of TNF-α, the higher level of pro-inflammatory cytokines might be due to the overall effects of oncogene activation. Apart from endogenous TNF-α, in our study, LPS and chemotherpeutic agents up-regulate TNF-α expression, indicating that TNF-α can act as a paracrine cytokine in malignancies.

Recently, we demonstrated that IL-6 is critical for lung cancer metastasis [11] and chemotherapeutic resistance [12]. Interestingly, our current data revealed that IL-6 could be well induced by TNF-α treatment (Supplementary Figure S7a-S7d). Moreover, IL-6 deficiency abrogated TNF-α's effects on cell migration (Supplementary Figure S7f-S7g) and MMPs expression (Supplementary Figure S7h), indicating that TNF-α regulate IL-6 expression [32–33]. Considering that IL-6 triggering ATM activation facilitate lung cancer metastasis via MMP-3/MMP-13 up-regulation, the above data suggest that TNF-α promoting lung cancer metastasis mostly, if not all, is depend on the up-regulation of IL-6.

ATM, an upstream regulator of NF-κB, play a critical role in IL-6 increasing lung cancer metastasis and chemotherapeutic resistance [11–12]. Erk1/2 and p38 were also documented to be up-stream regulators of NF-κB signaling in response to TNF-α treatment [34–38]. In this study, ATM inhibition abolish the effect of TNF-α on ERK/p38-NF-κB activation, unveiling that ATM is a key up-stream signal molecule in NF-κB activation. Further exploration is needed to focus on the cross-talk among inflammatory mediators and cellular effectors, which will insight the mechanism of initiation and development of inflammation associated cancers.

**Figure 8:**
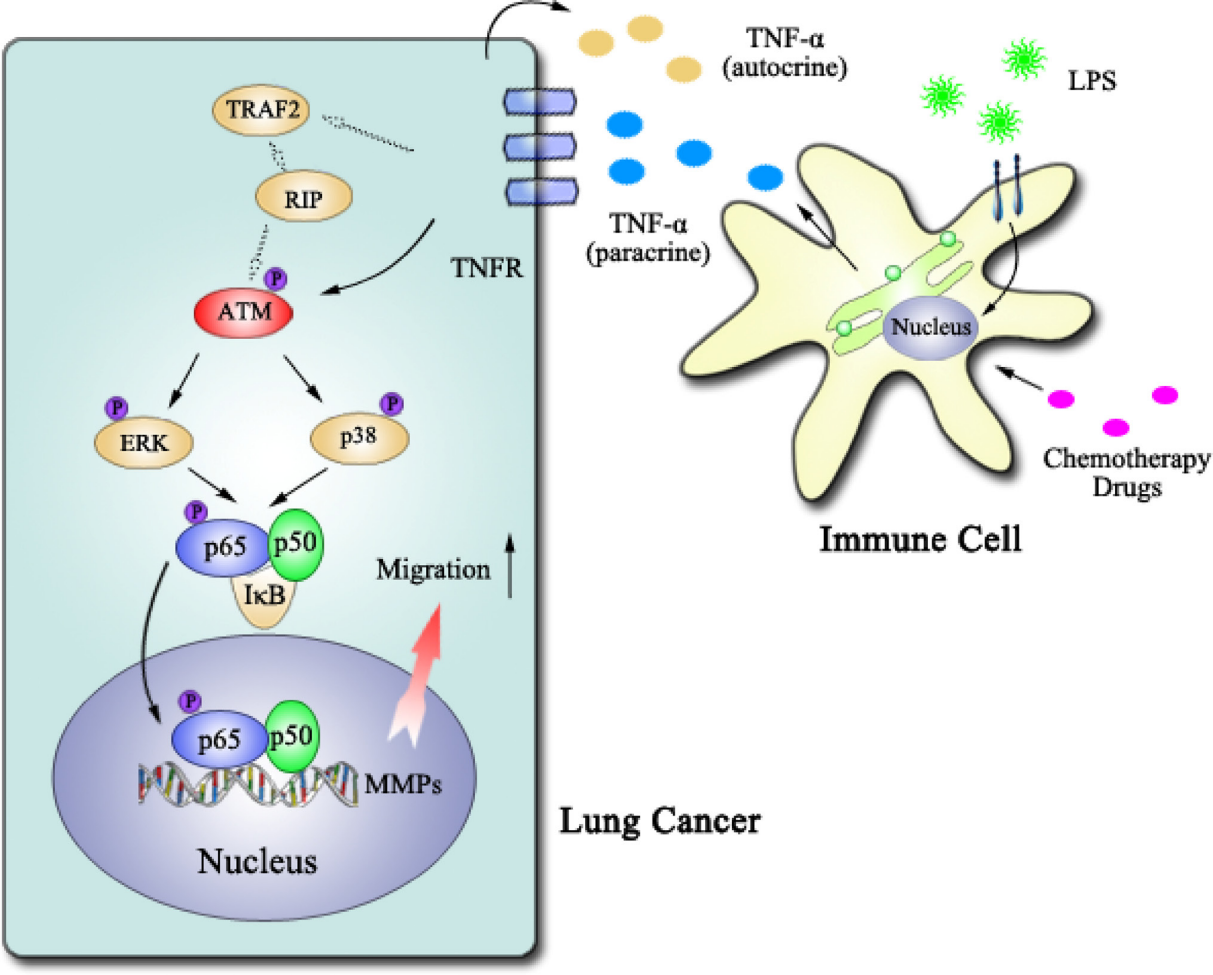
The model of ATM activation promoting lung cancer metastasis ATM, which could be activated by TNF-α in autocrine or paracrine manner, up-regulate MMP-13 expression via ERK/p38-NF-κB pathway and thereby augment lung cancer metastasis.

We therefore propose a novel mechanism for TNF-α augmented lung cancer cell migration. In this model, ATM is the key up-steam regulator of TNF-α activated ERK/p38-NF-κB pathway, which play a vital role in promoting lung cancer cell migration by up-regulating MMP-13 expression (Figure 8). Inactivation of TNF-α-ATM-ERK/p38-NF-κB decrease lung cancer metastasis, providing advanced evidences for ATM inhibitor usage in lung cancer treatment.

## MATERIALS AND METHODS

### Reagents

Recombinant human TNF-α, brefeldin A solution (BFA), human TNF-α Module Set ELISA kit were purchased from eBioscience (San Diego, CA, USA). Lipopolysaccharide (LPS) was obtained from Sigma-Aldrich (St. Louis, MO, USA). Secondary antibodies were obtained from Abcam (San Francisco, CA, USA). U0126, BAY11-7082 and all the primary antibodies were purchased from Cell Signaling Technology (Beverly, MA, USA). DAPI was obtained from Vector Laboratories, Inc. (Burlingame, CA, USA). Human PBMC isolation reagent was from Haoyang Biological Manufacture Co., Ltd. (Tianjin, China). SB203580 was acquired from Cayman Chemical (Ann Arbor, MI, USA). CGK733, Z-Pro-Leu-Gly-hydroxamate, 3B-3CT and NNGH were acquired from Sigma (Shanghai, China). UK-356618 was from Toronto Research Chemicals (North York, Canada). Pomalidomide was obtained from Selleck (Houston, TX, USA). All the small interfering RNAs and controls were purchased from Santa Cruz Biotechnology (Dallas, TX, USA). GM-CSF and IL-4 were obtained from R&D (Minneapolis, USA). Cisplatin (DDP) and camptothecin (CPT) were purchased from Calbiochem (San Diego, CA, USA). SYBR^®^ Premix Ex Taq™, Trizol and Prime Script reverse transcriptase were purchased from Takara (Dalian, China). Transwell compartment was from Corning (New York city, NY, USA). RPMI-1640, DMEM and fetal bovine serums were acquired from Hyclone (Logan, UT, USA). The immunohistological analysis was performed by using the UltraSensitiveTM SP IHC Kit (Fuzhou Maixin Biotech, china). Human MMP-3 and MMP-13 ELISA kits were from Shanghai Huiying Biotlogical Technology (Shanghai, China). Lipofectamine 2000 was bought from Invitrogen (Eugene, OR, USA).

### Animals

Pathogen-free C57BL/6 or BALB/c nude mice (female, 6-8 weeks old) were bought from Shanghai Laboratory Animal Center of Chinese Academy of Sciences (China) and kept at the Animal Center of Xiamen University. The protocol was approved by the Committee on the Ethics of Animal Experiments of the Xiamen University.

### Cell culture and cell lines

Human small cell lung cancer (SCLC) NCI-H446 cells, non-small cell lung cancer (NSCLC) NCI-H1299 cells, lung adenocarcinoma LTEP-a-2 cells and squamous cell carcinoma NCI-H520 cells and Raw264.7 cells were obtained from Type Culture Collection of the Chinese Academy of Sciences (Shanghai, China). Human lung carcinoma A549 cells were kindly provided by Professor GH. Jin (Xiamen University). All the cells were grown in RPMI-1640 or DMEM medium containing 10% FBS.

### siRNA interference

Most of the small interfering RNAs and controls were purchased from Santa Cruz Biotechnology. They are TNF-α siRNA (sc-37216), ATM siRNA (sc-29761), NF-κB p65 siRNA (sc-29410), hMMP3 siRNA (sc-29399), hMMP9 siRNA (sc-29400), hMMP13 siRNA (sc-41559) and control siRNA (sc-37007). p38 MAPK siRNA (#6564) and p44/42 MAPK (Erk1/2) siRNA (#6560) were obtained from Cell Signaling Technology. For siRNA transfection, 30-50% confluent cells were transfected with siRNA using Lipofectamine 2000. The cells were harvested 48 h after transfection. The final concentration for siRNA is 100 nM. The silence effects of indicative siRNA in NCI-H446 cells were validated in Supplementary Figure S8.

### Bone marrow-derived murine DCs

Bone marrow-derived immature dendritic cells (imDCs) were prepared as previously described [39]. Briefly, bone marrow mononuclear cells were prepared from bone marrow suspensions of C57BL/6 mice by depletion of red cells and then were cultured at a density of 1×10^6^ cells/ml in RPMI 1640 complete medium with 10 ng/ml of GM-CSF and 1 ng/ml of IL-4. Non-adherent cells were gently washed out on day 4 of culture; the remaining loosely adherent clusters were used as im-DCs and were further stimulated with LPS, DDP or CPT.

### Murine splenocytes preparation

Spleens isolated from C57BL/6 mice were washed in PBS and ground into single cells. Then the cell suspension was re-suspended followed by depletion of red cells and cultured at a density of 1×10^6^ cells/ml in RPMI 1640 medium for further treatment.

### Preparation of human peripheral mononuclear cells (PBMCs)

Briefly, human PBMC was prepared from the donor's blood by gradient density centrifugation using PBMC isolation reagents and cultured at a density of 1×10^6^ cells/ml in RPMI 1640 medium for further treatment. Healthy subjects were selected according to the Declaration of Helsinki principles and signed an informed consent.

### RT-qPCR

Endogenous TNF-α expression of lung cancer cells and MMPs expression were investigated by RT-qPCR analysis as previously described [11] The PCR primer sequences were described in Supplementary Table S1.

### ELISA

To detect the release of TNF-α or IL-6 in corresponding lung cancer cell lines and determine MMP-3/MMP-13 activity, enzyme double-antibody indirect immunoassays with respective ELISA kits was performed in accordance with manufacturer protocol [40].

### Western blot

Cells were treated with TNF-α (2 ng/ml) for indicated periods or pretreated with inhibitors or siRNA prior to TNF-α stimulation. The expressions of indicated proteins were determined via western blot analysis. ß-actin was used as a loading control.

### Transwell migration assay

Cell migration ability was determined via transwell migration assay as previous description [11, 22].

### Confocal immunofluorescence assays

The effects of TNF-α or ATM siRNA on ATM, p65, ERK or p38 phosphorylation levels was investigated using immunofluorescence assays as previous description [12].

### Flow cytometric measurements

The effects of LPS, DDP or CPT on TNF-α expression were assayed by flow cytometric assays [12].

### *In vivo* lung cancer metastasis model

Cell migration ability was determined via murine lung cancer metastasis model [25]. Briefly, NCI-H520 cells transfected with control or ATM siRNA were inoculated into BALB/c nude mice through the tail vein. 27 days after the injection, the mice were sacrificed and the lungs were dissociated and preserved for further studies. Each experiment had three mice per condition.

### H&E and immunohistochemistry staining

*In vivo* cell migration ability and the expression of TNF-α in lung tissues were determined via H&E and Immunohistochemistry staining as described before [40].

### Statistical analysis

All experiments were repeated at least three times to confirm the similar results. Data were presented as the mean ± SEM. Student's *t* test or one-way ANOVA with the post Newman-Keuls test was applied. Statistical differences were considered to be significant at p<0.05.

## SUPPLEMENTARY FIGURES AND TABLE


