# Supplementary material for: Ataxia-telangiectasia mutated activation mediates tumor necrosis factor-alpha induced MMP-13 up-regulation and metastasis in lung cancer cells
Source: Oncotarget. 2016 Aug 19;7(38):62070–83. doi: 10.18632/oncotarget.11386 (PMC5308711; doi:10.18632/oncotarget.11386)
Supplement: Supplementary file 1 [file oncotarget-07-62070-s001.pdf]

Ataxia-telangiectasia mutated activation mediates tumor necrosis factor-alpha induced MMP-13 up-regulation and metastasis in lung cancer cells

SUPPLEMENTARY FIGURES AND TABLE

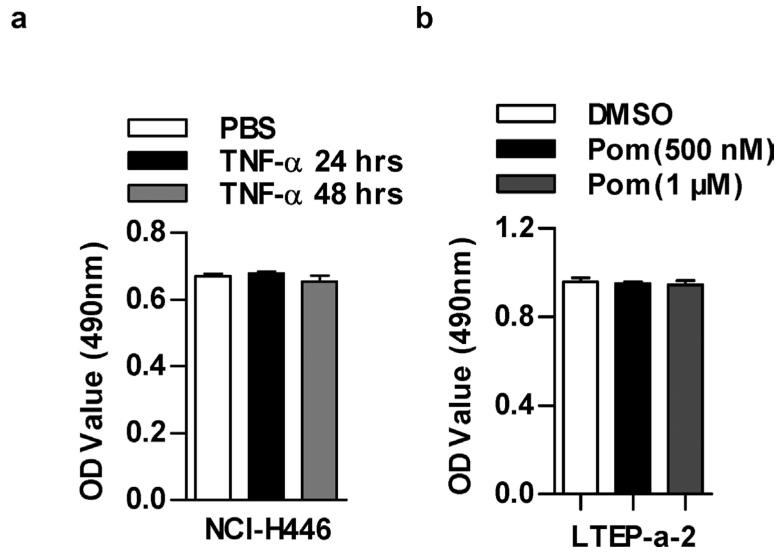

Supplementary Figure S1: The effects of TNF-α and pomalidomide on lung cancer cell viabilities. NCI-H446 **a.** or LTEP-a-2 **b.** cells were treated with TNF-α (2 ng/ml) (a) or TNF-α inhibitor Pom (b), respectively, for indicated period or concentration and cell viabilities were determined by MTT assay. Data are presented as the mean±SEM, n=3. One-way ANOVA with post Newman-Keuls test. Hours and pomalidomide was shown as Hrs or Pom for limited space in the figure.

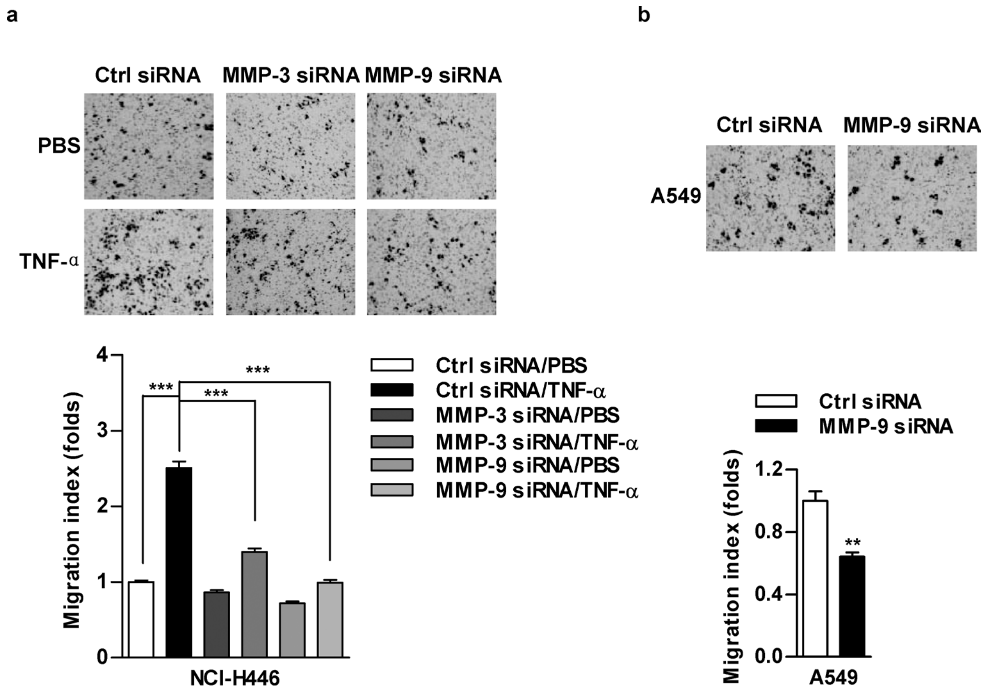

Supplementary Figure S2: MMP-3 and MMP-9 are involved in TNF-α increasing lung cancer cell migration. a. MMP-3 or MMP-9 deficient NCI-H446 and control cells were treated with TNF-α (2 ng/ml) and the effect of TNF-α on cell migration was determined by Transwell migration assay. **b.** A549 cells were conferred MMP-9 siRNA transfection and the cell migration was determined by Transwell migration assay. Data are presented as the mean±SEM, n=3. **p<0.01; ***p<0.001, Student *t* test or One-way ANOVA with post Newman-Keuls test. One representative from three experiments is shown.

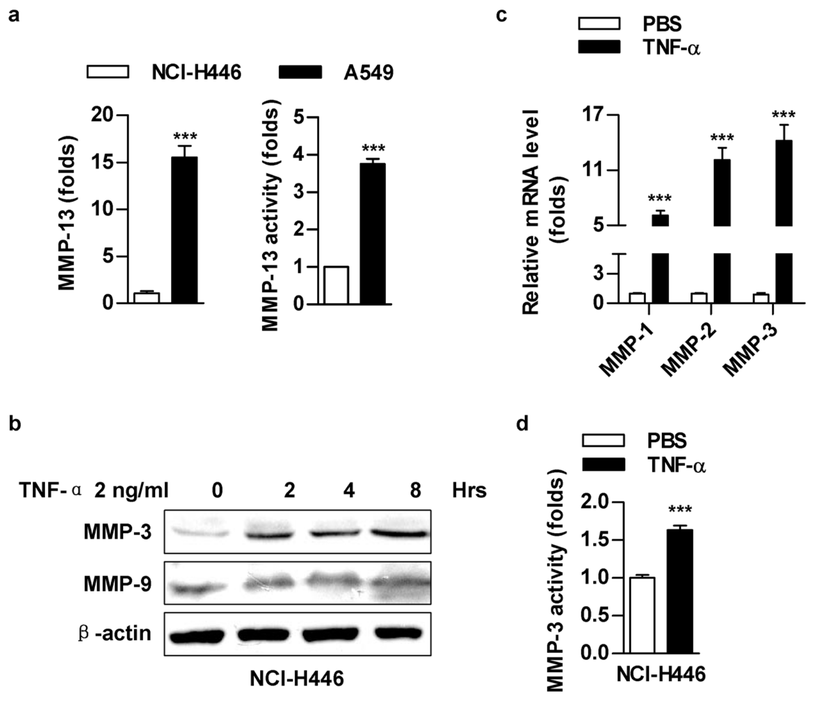

Supplementary Figure S3: TNF-α increases MMPs expression and activity in lung cancer cells. a. The cellular RNA and supernatant of NCI-H446 and A549 cells were prepared. The expression (left) and the activity (right) of MMP-13 was determined by RT-qPCR (left) or ELISA (right). **b-d.** NCI-H446 cells were treated with TNF-α (2 ng/ml) or control PBS. The expression (b-d) and activity (d) of indicated MMPs were determined by western blot (b), RT-qPCR (c) or ELISA (d), respectively. Data are presented as the mean±SEM, n=3. ***p<0.001, Student t test. One representative from three experiments is shown. The immunoblots were cropped to improve the clarity and conciseness of the presentation.

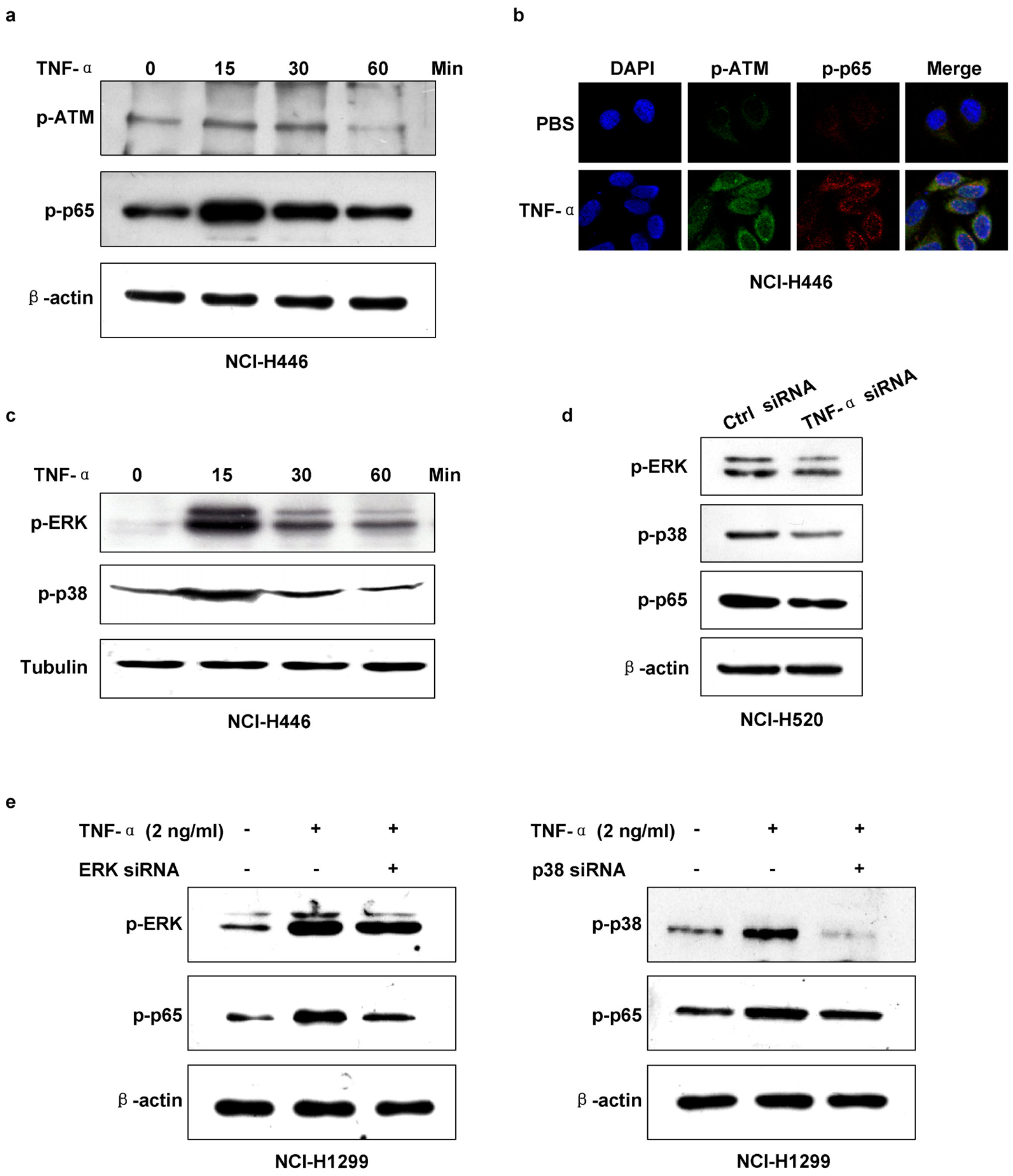

Supplementary Figure S4: TNF-α activates ATM-ERK/p38- p65 pathway. NCI-H446 cells **a-c.** were stimulated with TNF-α (2 ng/ml) for indicated periods while NCI-H1299 cells **d-e.** were conferred with indicated siRNA transfection, and the effect of TNF-α on related kinases were determined by western blot (a, c-e) and immunofluorescence observation (b). The immunoblots were cropped to improve the clarity and conciseness of the presentation.

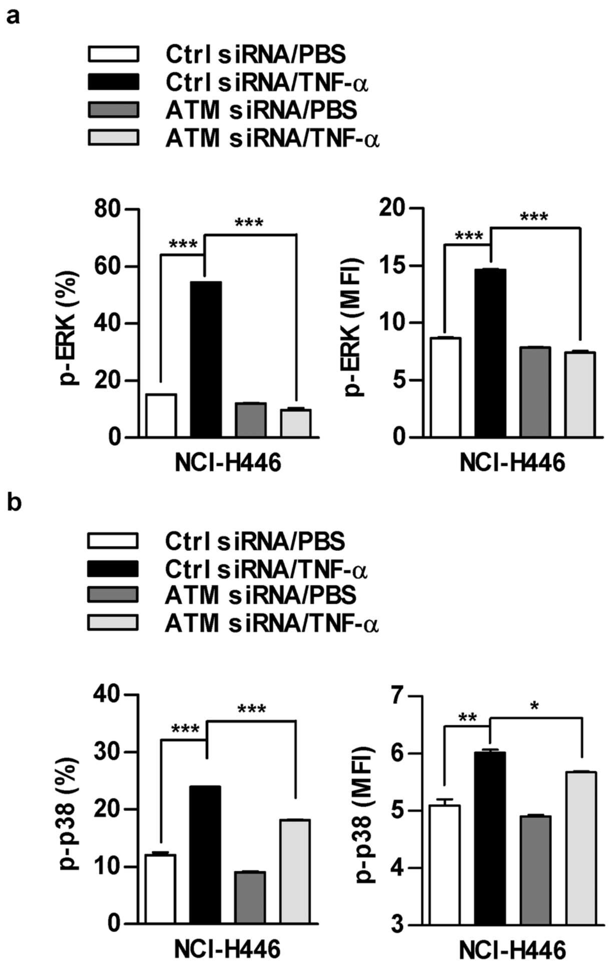

Supplementary Figure S5: The deficiency of ATM decreases TNF-α-induced phosphorylation of ERK and p38. NCI-H446 cells were conferred ATM siRNA transfection and further stimulated with TNF-α (2 ng/ml). The effects of TNF-α on ERK **a.**/ p38 **b.** activation in ATM deficient NCI-H446 cells was determined by flow cytometric analyses. Data are presented as the mean±SEM, n=3. *p<0.1; **p<0.01; ***p<0.001, One-way ANOVA with post Newman-Keuls test. One representative from three experiments is shown.

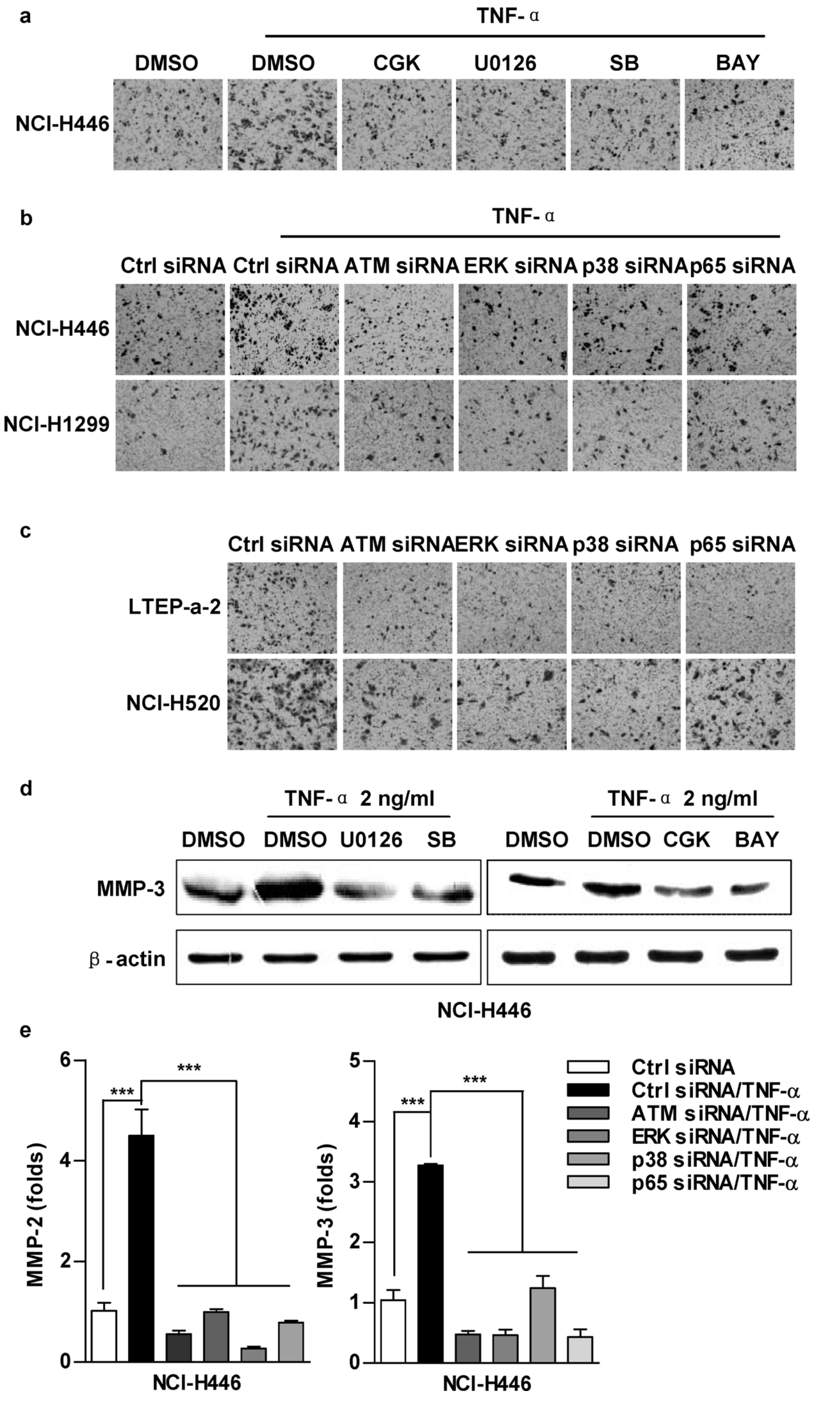

Supplementary Figure S6: TNF-α up-regulate cell migrationg abilities and MMP-2/MMP-3 expression via ATM-ERK/p38-NF-κB pathway. a-c. The migration images of Fig 5a-5c were shown, respectively. **d-e.** NCI-H446 cells were pretreated with indicated kinase inhibitors (d) or siRNA transfection (e) prior to TNF-α (2 ng/ml) stimulation. The effect of kinase inhibition on indicated MMPs expression was determined by western blot (d) and RT-qPCR (e), respectively. Data are presented as the mean±SEM, n=3. ***$p<0.001$, One-way ANOVA with post Newman-Keuls test. One representative from three experiments is shown. CGK733, SB203580 and BAY11-7082 were shown as CGK, SB and BAY, respectively, for limited space in the figure. The immunoblots were cropped to improve the clarity and conciseness of the presentation.

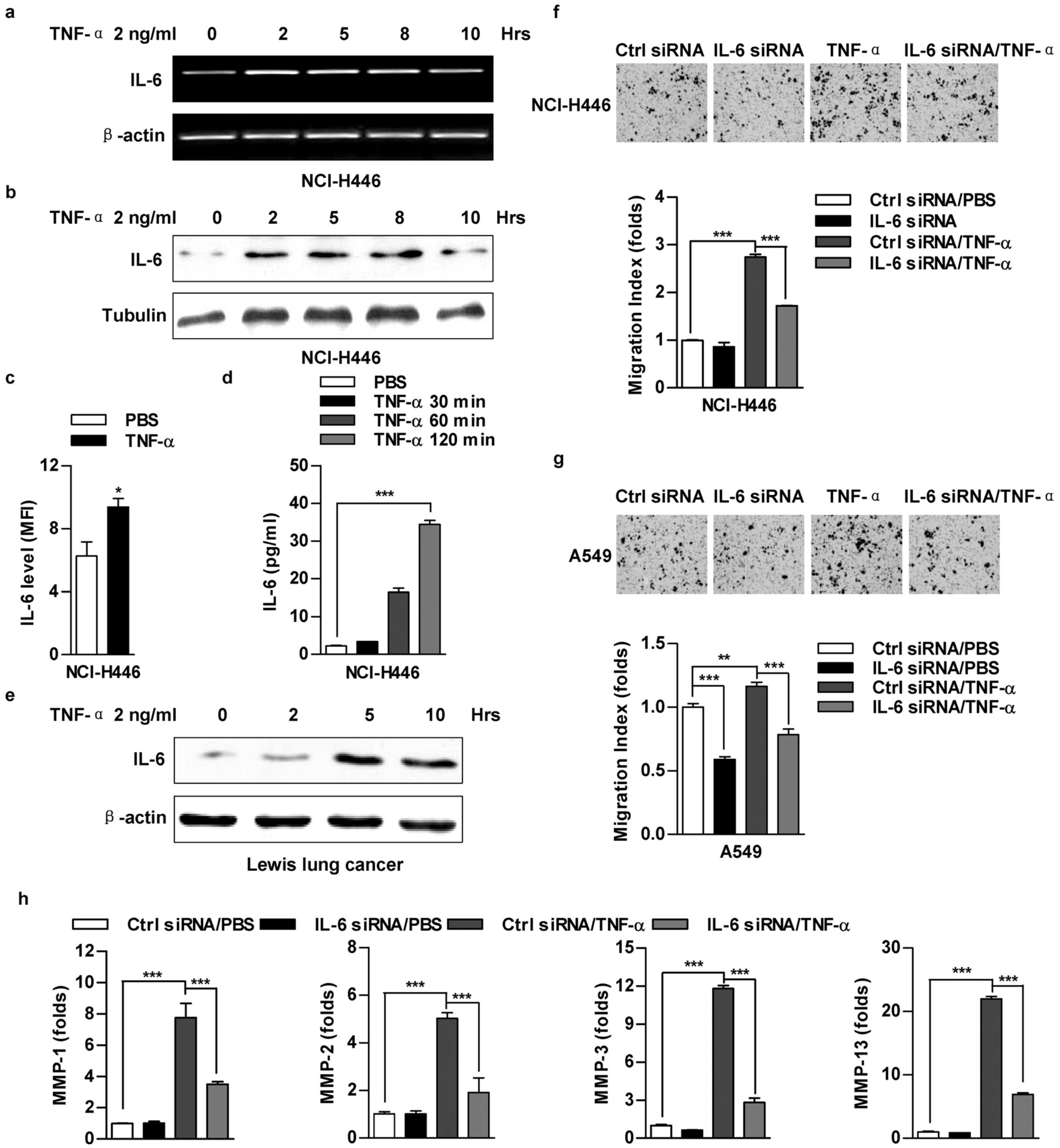

Supplementary Figure S7: IL-6 is involved in TNF-α-increased MMPs expression and thereby augmented migration ability of lung cancer cells. a-e. NCI-H446 (a-d) or Lewis lung cancer cells (e) cells were treated with TNF-α (2 ng/ml) for indicated periods and the expression of IL-6 was detected by RT-PCR (a), Western blot (b, e), flow cytometry (c) or ELISA assay (d). **f-h.** IL-6 deficient and control NCI-H446 (f, h) or A549 cells (g) were treated with TNF-α (2 ng/ml), and the effect of IL-6 deficiency on TNF-α-induced cell migration (f-g) and MMPs expression (h) were determined by Transwell migration assay (f-g) and RT-qPCR (h), respectively. Data are presented as the mean±SEM, n=3. **p<0.01; ***p<0.001, Student t test or one-way ANOVA with post Newman-Keuls test. One representative from three experiments is shown. The immunoblots were cropped to improve the clarity and conciseness of the presentation.

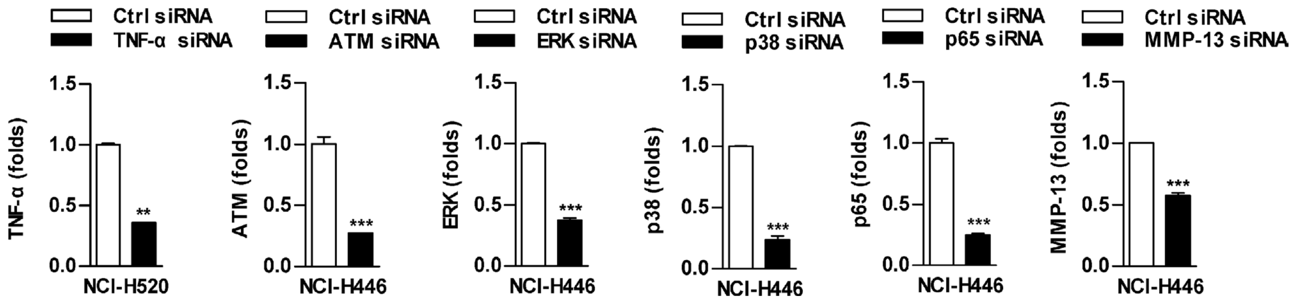

Supplementary Figure S8: siRNA transfection efficiently decreased relative gene expression in lung cancer cells. NCI-H520 or NCI-H446 cells were conferred indicated siRNA transfection and the expressions of indicated gene were determined by RT-qPCR. Data are presented as the mean±SEM, n=3. **p<0.01; ***p<0.001, Student *t* test. One representative from three experiments is shown.

Supplementary Table S1: Real-time PCR primers and small interference RNA sequences

Genes	F/R	Sequence
β-actin	F	5'-TCAAGATCATTGCTCCTCCTG-3'
β-actin	R	5'-CTGCTTGCTGATCCACATCTG-3'
Human TNF-α	F	5'-CAGCCTCTTCTCCTTCCTGAT-3'
Human TNF-α	R	5'-GCCAGAGGGCTGATTAGAGA-3'
MMP-13	F	5'-TTGTTGCTGCGCATGAGTTCG-3'
MMP-13	R	5'-GGGTGCTCATATGCAGCATCA-3'
Mouse TNF-α	F	5'-ATGAGCACAGAA AGCATGATC-3
Mouse TNF-α	R	5'-TACAGGCTTGTCACTCGAATT-3'
MMP-1	F	5'-GGAGGGGATGCTCATTTTGATG-3
MMP-1	R	5'-TAGGGAAGCCAAAGGAGCTGT-3'
MMP-2	F	5'-CCCCAAAACGGACAAAGAG-3'
MMP-2	R	5'-CTTCAGCACAAACAGGTTGC-3'
MMP-3	F	5'-CCTGCTTTGTCCTTTGATGC-3'
MMP-3	R	5'-TGAGTCAATCCCTGGAAAGTC-3'
